# Judicialization of health: profile of demands for oncological medicines in a state in the central region of Brazil

**DOI:** 10.1186/s12939-022-01704-6

**Published:** 2022-08-17

**Authors:** Leila Abou Salha, Flávia Costa Reis, Roberta Moreira Gonçalves, Jordão Horácio da Silva Lima, Nádia Abou Salha, Roney Pereira Pinto, José Elmo de Menezes, Eduardo Perez Oliveira, Pedro Lopes Ferreira, Maria Alves Barbosa

**Affiliations:** 1 Faculty of Medicine, Federal University of Goiás, Goiania, Goiás 74605-050 Brazil; 2 School of Law, Business and Communication, Pontifical Catholic University of Goiás, Goiania, Goiás 74805-100 Brazil; 3 University of São Paulo, São Paulo, 01246-904 Brazil; 4 Physician, Petrópolis Medical School, Petrópolis, Rio de Janeiro, 25680-120 Brazil; 5 State Health Department, Center for High-Cost Medicines Juarez Barbosa, Goiânia, Goiás 74015-020 Brazil; 6 Federal Institute of Education, Science, and Technology of Goiás, Pontifical Catholic University of Goiás, Goiania, Goiás 74605-900 Brazil; 7 Judge, Court of Justice of the State of Goiás, Goiânia, Goiás 74130-011 Brazil; 8 Center for Health Studies and Research, Faculty of Economics, University of Coimbra, 3004‐512 Coimbra, Portugal

**Keywords:** Judicialization of health, Antineoplastic Agents, Quality of life

## Abstract

**Background:**

The significant increase in access to oncological medicines through court cases suggests that constitutional guarantees of integral and universal care in the Brazilian public health system are uncertain.

**Methods:**

A retrospective observational study was conducted to analyze data from lawsuits requesting oncological medicines from 2014 to 2020 in the State of Goiás, Brazil, in state and federal courts. Sociodemographic, medical, and legal variables were statistically examined using descriptive, association, and correlation methods.

**Results:**

Women brought more than half (54%) of the 301 processes analyzed. The most frequent age group was over 55 years, with income below 3 × the minimum wage (total about USD$600/month), and their cases were promoted through the public minister and public defender’s offices. The most requested medications, not on official public health system lists, were indicated for multiple myeloma and brain cancer.

**Conclusions:**

Improved quality of life, frequently used as a justification, could be conceptually confused with increased survival. Finally, judicialization itself indicates that individual health needs arise even with properly defined and adequately implemented public policies. These needs should be considered for the adequate provisioning of services by the state to ensure the right to health.

## Introduction

Health care is a constitutional right in Brazil, and article 196 of the constitution states that health is a “right of all and also a duty of the State, guaranteed through social and economic promotion, protection, and recovery policies aimed at reducing the risk of disease and guaranteeing access to services” [1].

The Brazilian government is organized into three harmonious and independent branches, executive, legislative, and judicial, with typical and atypical executive, legislative and jurisdictional functions. In addition, the essential functions of justice are the Public Prosecutor’s Office *(Ministério Público)* and the Public Defender’s Office *(Defensoria Pública)*, which operate independently in the states and from the Union. The Executive branch is responsible for managing the public health system—*SUS*, as the executor of constitutional guidelines to guarantee integral healthe care [1].

Brazil is one of about a hundred countries recognizing the constitutional right to health, emphasizing comprehensive pharmaceutical care [2]. In addition, using a judicial process to access health services also occurs in other countries, such as Chile, Colombia, and Costa Rica [3–5]. However, improving the quality of medications offered in *SUS* and ensuring access to medications presents several challenges, such as increased life expectancy and the aging population, the demand of patients for treatments to be included, and offering health interventions [6]. These challenges contribute to the process of “medicationization” or “medicalization” [7], or even “pharmaceuticalization” [2] when medications are used in situations that cannot be considered diseases or the effects are overestimated. These processes are closely linked to the current economic-social model, where the use of medicines in society is separate from scientific medical-health criteria and rational use [8].

Through *SUS*, universal access to comprehensive health care was provided, aiming at prevention and health promotion, focused on quality of life for the population [9]. Quality of life refers to an individual’s perception of essential aspects of their standard of living, such as their physical health, vitality, social relationships, the environment they are inserted into, emotional health, and spirituality. In addition, with chronic diseases, such as cancer, quality of life is a predictor of morbidity and mortality and a measure for evaluating treatment effects [10–12].

Through a presidential decree in 2000, the Executive branch designated the National Cancer Institute (*INCA*) as the formulator and developer of policies to prevent and control cancer in the country [13, 14]. Furthermore, to integrate resources to prevent and fight cancer, the federal government created the national cancer care support program *(PRONON)* in 2012 [15]. PRONON consists of the organization of lines of care at all levels, including promotion, prevention, diagnosis, rehabilitation, and palliative care [16].

The *SUS* pharmaceutical service in basic, strategic, and specialized components does not include the supply of oncologic drugs [17]. Moreover, *SUS* has no single list of antineoplastic drugs, and oncology clinical guidelines contemplate only some types of cancer [18]. The *SUS* path to oncological treatment, including drugs and other health technologies, is unique: An individual diagnosed with a neoplasm must be referred to a High Complexity Oncology Care Unit *(UNACON)* or a High Complexity Oncology Care Center *(CACON)*, institutions linked to the Ministry of Health, distributed throughout the country to treat various types of cancer [19, 20]. Currently, there are 317 qualified care units and care centers in the country, with five units in the state of Goiás [21, 22].

Pharmaceutical care in cancer treatment is specially designated as oncological care and is included in medium and high-complexity health care funding. Cancer treatment is reimbursed by specific procedures (surgical, radiotherapy, chemotherapy, and iodotherapy), depending on the stage of the disease, histological type, treatment objectives (curative or palliative), and the therapeutic means to be used [17]. *SUS* does not restrict or establish a specific chemotherapy drug. That is, reimbursement does not depend on the therapeutic regimen. Thus, the responsibility for acquiring, standardizing, and supplying chemotherapy drugs belongs to the qualified public or private institution (*UNACON* or *CACON*) [23, 24].

In Brazil, structural limitations create obstacles to financing the health care system through public–private incentives. Therefore, Brazil is the only country with a comprehensive and universal health system with higher private spending than public spending [25, 26].

Not many studies address oncology in Brazil. However, public spending on medicines impacts *SUS,* and a large part of these costs is due to advanced-stage treatment, requiring more procedures and medications. Therefore, early diagnosis is promoted to save lives and minimize the financial impact on public health expenditures [27].

Controlling environmental risk factors and lifestyle changes can reduce the incidence of new cancer cases, and in developed countries, the reduction can vary from one-third to two-fifths [28–30]. Despite the importance of prevention with proven effective interventions, by reducing exposure to known causes of human cancer or by interrupting the progression in various stages of tumors, national and international policymakers still need encouragement to recognize the benefits and advances achieved [30, 31].

From 2010 to 2015, there was a 66% increase from R$2 billion to R$3.5 billion (1 BRL is about 5 USD), including surgical procedures, chemotherapy, radiotherapy, hormone therapy, and palliative care. There was also an increase in the number of individuals undergoing treatment, from about 292,000 to about 393,000 [32]. In 2017, public spending on treatments (chemotherapy, radiotherapy, and hormone therapy) totaled R$4.5 billion reais, 25% of total expenses. In 2018, expenses were 4.6 billion reais, and chemotherapy alone was responsible for 49% of expenses. On average, each individual with cancer cost *SUS* around R$9000 in 2018 [33].

In 2019, the total monthly burden on the individual in cancer treatment and their caregiver was, on average, from R$290 to R$300, indicating a high financial impact on the average Brazilian family income [25].

Health expenditures represent 8.2% of the GDP. However, less than 50% of this total is for the cost of the public system [34]. Per capita spending on health in 2017 was comparatively higher than in Latin American countries, such as Colombia, but three times lower than in OECD countries [35]. The latest data from the OECD show that Brazil’s 2019 health expenditure was 9.59% of GDP. The breakdown of health expenditure by type of financing is distributed as follows: public (40%); voluntary health insurance (30%); direct out-of-pocket expenses (25%); other (5%) [36].

*SUS* has been underfunded since it was created. For example, social welfare transfers were interrupted in 1993, and part of the health budget provided by the Social Emergency Fund was de-funded when federal revenues were unlinked. Later, the CPMF (a tax on financial transactions) was intended to subsidize public health expenses, resulting in the government adding additional budgetary items to the Ministry of Health. Federal funding of *SUS* through Constitutional Amendment 29 (EC-29) took eight years and allocated perpetually insufficient resources to *SUS*, further complicated by the approval of Constitutional Amendment 86 in 2015 [37, 38].

Moreover, the approval of Constitutional Amendment 95 (EC-95) in 2016 freezes the federal government’s primary expenditure and its minimum application in health actions and services, in real terms, until 2036 and represents an obstacle to the expansion of SUS funding by preventing the allocation of more resources to health without de-funding other policies at the federal level [39] De-funding estimates approached R$22.5 billion between 2018 and 2020 [40].

It is important to note that the inclusion of health as a fundamental constitutional right was not enough to guarantee it [41]. Indeed, these medications are not always available, and this is especially relevant to the current topic because the patient must then resort to judicial intervention to safeguard their constitutionally protected right [42]. As the right to health is a subjective fundamental right, the legal system allows this right to be asserted in the judiciary [1].

Judicialization of health refers to an intervention in Public Administration by the judiciary to guarantee the constitutional right to care, such as provisioning medications and products and services [43, 44] or even mediating the supply–demand relationship to secure constitutional guarantees [45].

Despite all the regulations on each political entity that implements pharmaceutical care, the judiciary has come to understand that federal entities have a joint responsibility to provide medications and health treatments [13, 46]. However, only the federal government must be sued when it comes to medications that are not registered with the National Health Surveillance Agency (*ANVISA*) [47].

Data made available by the National Council of Justice reveal that the supply of medicines is the leading cause of litigation against *SUS*, indicating that the issue requires the coordinated action of all health and justice actors [48]. However, judicialisation is not something new. The first lawsuits were filed in 1990 requesting medication for HIV/AIDS treatment, and since then, the judiciary has become the means for acquiring high-cost drugs for more diverse treatment options [49].

There is no consensus on the definition of high-cost medication. However, some conceptual approximations have been made using qualitative indicators like medicines with high health risks and quantitative indicators like regulating the cost of treatment. Others still use it as a synonym for highly complex and costly medicines, with precise use conditions and expensive medicines from limited sources [50].

Judicialization is already institutionalized as an alternative form of access to new technologies through *SUS* [51]. However, it is undeniable that this avenue of entry often violates the right to equality in a community that is supposed to be served by *SUS* [52].

The expansion of adequate public health services depends on the commitment of the State and its leadership [53]. Many of these lawsuits seek to ensure patients’ right to high-cost medicines that are not always available in *SUS* and often without proven benefits, or even, in some cases, with deleterious/side effects and adverse impacts on quality of life [33]. The Judiciary has self-compositional methodologies for conflict resolution from the Judicial Centers for Conflict and Citizenship Solutions (CEJUSC), in addition to the possibility of opinions from health professionals by the Judiciary Technical Support Center (NATJUS) as subsidies for the judicial decision [54, 55].

From 2015 to 2020, there were more than a million new lawsuits concerning medicines in Brazil. In 2020, this figure included 196,000 new lawsuits, most seeking new drugs not provided by *SUS* or private insurance [41].

The imbalance in SUS pharmaceutical care resulting from the judicialization of oncological medicines has a high impact due to the high cost, the complexity of the treatment, and pressure from the pharmaceutical industry [56].

The phenomenon of judicialization can be understood by analyzing relevant aspects of lawsuits and their impact on quality of life to help mitigate the phenomenon and fill the gap in broader studies on the subject in the Midwest Region of Brazil. Therefore, the study’s objective was to analyze the results of lawsuits on requests for oncological drugs in the State of Goiás from 2014 to 2020.

## Method

A retrospective analytical study was conducted from July to October 2021, with data obtained from individual lawsuits requesting high-cost cancer drugs at the Goiás State Court of Justice (TJGO) and the Federal Regional Court – Goiás Section (TRF-Goiás).

The present study is part of a larger project entitled: Quality of life of oncological drug claimants in administrative and judicial proceedings in Goiás.

The data were obtained from the Technical Analysis Sector of the Central de Medicamentos de Alto Custo Juarez Barbosa / Center for High-Cost Medications (*CEMAC*) through the registration of oncological medicines and the respective users in the judicial process of granting the supply. In addition, the lawsuits were also checked in the judicial demand protocol system of *TJGO* (Digital Judicial Process—*PROJUDI*) and *TRF*-*Goiás* (Electronic Judicial Process—*PJe*) for confirmation and double-checking.

*CEMAC* is the referral center in the state of Goiás, Brazil, for dispensing drugs from the Specialized Pharmaceutical Service Component (*CEAF*) located in the city of Goiânia, Goiás. The list of individuals registered to receive oncological medicines was obtained in the Pharmacy Sector of the Judiciary. The judicial processes were accessed in the Technical Analysis Sector of *CEMAC*, where two researchers with law qualifications collected the data presented in this study. The data obtained were confirmed in the procedural search in *PROJUDI* and *PJe*.

All current processes that requested oncological medicines were included. The process initiated from 2014 to 2020. Duplicate processes at *TJGO* and *TRF*-Goiás and processes requesting medical or nutritional products, procedures, medical consultations, surgery, and diagnostic tests were excluded.

The study variables were based on the Manual of Indicators for the Evaluation and Monitoring of Judicial Demands for Medicines of the National School of Public Health/*FIOCRUZ* [57]. They were separated into 3 groups: sociodemographic, medical-health, and legal. The sociodemographic variables were gender, age, income, education, and declared occupation. The medical-health variables were the ICD-10 [58], name and quantity of the medication requested, the origin of the prescription, and the origin of the medical report (public or private). Finally, the legal variables were the promotor or office that filed the action (Public Prosecutor, Public Defender, or Private Lawyer), dates of filing, preliminary decision or anticipation of protection and first-degree ruling, request for expertise, presence of *NATJUS* or *CEJUS* opinion and justifying the request using quality of life.

The statistical analysis consisted of descriptive statistics with a distribution of simple and relative frequencies and analysis of measures of central tendency (mean, mode and median), and respective analyzes of variability or dispersion measures (amplitude, interquartile range, variance, standard deviation, minimum and maximum). Microsoft® Excel 2019 was used for data tabulation. Statistical Package for Social Sciences software (SPSS® Windows® version 28.0.1, IBM Company, Chicago, IL) was used for data analysis. To evaluate the legal variables and the relationship with the variables of the sociodemographic profile with more than two groups, variance analysis (ANOVA) was used, and for the other cases, Student’s t-test was used. In addition, Pearson’s correlation coefficient was calculated for comparative detailing. Finally, a statistical significance of (*p* < 0.05) was used for all tests.

The research involved data from human beings and complied with the National Health Council according to Resolutions No. 466/2012 and No. 510/2016. It was approved on August 20, 2018, by the Research Ethics Committee of the Federal University of Goiás, Brazil, under opinion no. 2.831.905 and no 4.558.046 – CAAE 93,238,318.7.0000.508351–52 [59, 60], part of the project entitled Quality of life of cancer drug claimants in administrative and judicial proceedings in Goiás.

## Results

**Fig. 1:**
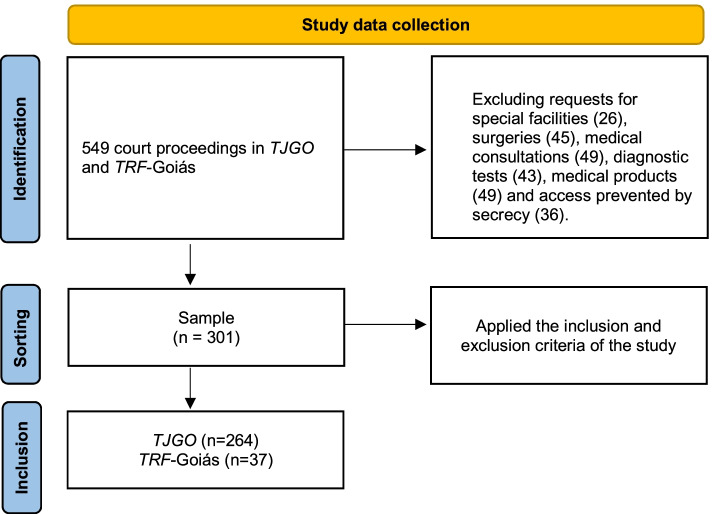
Study data collection flowchart. Goiania, 2020. Source: Study data

This study identified 549 cases in the study period; 301 met the inclusion criteria and were analyzed. The excluded cases were related to requests for special facilities (26), surgeries (45), medical consultations (49), diagnostic tests (43), medical products (49), and cases where access was prevented by judicial secrecy (36). Of the 301 cases analyzed, 264 (88%) were filed in the state courts (*TJ*) and 37 (12%) in the federal courts (*TRF(GO)*) (Fig. 1).

All actions were individual. The complainants were primarily women (54%). The predominant age bracket was 65 years or more (30%). However, most were in the age bracket above 55 years (52.5%). The occupations described were diverse, highlighting retirees (27%) and domestic workers (11%). The family income, when declared, was up to 3 minimum wages (33%). Notably, none of the cases analyzed reported a family income higher than 3 minimum wages (Table 1).

**Table 1:** Sociodemographic profile of participants (complainants)

Variables	All groups		TJ		TRF (GO)	
	n	%	n	%	n	%
**Gender**	301	100%	264	88%	37	12%
Male	138	46%	123	41%	15	5%
Female	163	54%	141	47%	22	7%
**Age**	301	100%	264	88%	37	12%
0–17	10	3%	8	3%	2	1%
18–24	8	3%	6	2%	2	1%
25–34	25	8%	21	7%	4	1%
35–44	41	14%	39	13%	4	1%
45–54	59	20%	50	17%	7	2%
55–64	67	22%	86	29%	7	2%
65 years or older	91	30%	54	18%	11	4%
**Occupation**	301	73%	264	88%	37	12%
Retiree	81	27%	70	23%	11	4%
Social Assistance or pensioner	6	2%	5	2%	1	0%
Student	5	2%	4	1%	1	0%
self-employed professional	28	9%	26	9%	2	1%
Rural worker	11	4%	10	3%	1	0%
domestic worker	32	11%	30	10%	2	1%
Others	138	46%	119	40%	19	6%
**Family income**^a^	301	100%	264	88%	37	12%
Less than 1 SM (up to USD 209)	48	16%	43	14%	5	2%
1–3 SM (between USD 209–627)	51	17%	40	13%	11	4%
Above 3SM (more than USD 627)	0	0%	0	0%		0%
Not Specified	202	67%	181	60%	21	7%

^a^Family income: The national minimum wage (SM) in 2020 was R$1045.00, 1USD = (apx) R$5.00 [61]

The number of cases in the *TRF(GO)* was comparatively lower than *TJ*. Table 1 also shows a majority of women and retirees in both channels, with a difference in the predominant age groups, being > 55 years in the *TJ* and > 65 years in TRF(GO), and family income, being in the *TJ* < 1 minimum wage and *TRF(GO)*, between 1 and 3 minimum wages.

The most requested drugs were temozolomide (14%) and bevacizumab (13%), followed by rituximab (9%), pazopanib (8%), ibrutinib (5%), pembrolizumab (5%) and bortezomib (3%). Regarding the ICD-10 [58], the most frequent codes used were C71 (malignant neoplasm of brain) and C90 (multiple myeloma and malignant plasma cell neoplasms) (Table 2).

**Table 2:** Medical and health variables of the study

Variables	All groups		TJGO			TRF (GO)		
	n	%	n	%	n			%
**Drug**	301	100%	264	88%	37			12%
temozolamide	42	14%	41	14%	1			0%
bevacizumab	39	13%	33	11%	6			2%
rituximab	26	9%	24	8%	2			1%
pazopanib	25	8%	18	6%	7			2%
pembrolizumab	15	5%	15	5%	0			0%
ibrutinib	15	5%	14	5%	1			0%
bortezomib	10	3%	9	3%	1			0%
Others	129	43%	110	37%	19			6%
**ICD-10**^a^	301	100%	264	88%	37			12%
C-71malignant neoplasm of brain	40	13.3%	39	13%	1			0.3%
C-90 multiple myeloma and malignant plasma cell neoplasms	38	12.7%	33	11%	5			1.7%
C-53 malignant neoplasm of cervix uterin	28	9.3%	24	8%	4			1.3%
C-64 malignant neoplasm of kidney, except renal pelvis	26	9%	20	7%	6			2.0%
C-43 malignant melanoma of skin	15	5%	15	5%	0			0.0%
C-91 lymphoid leukemia	15	5%	12	4%	3			1.0%
C-18 malignant neoplasm of colon	13	4.3%	12	4%	1			0.3%
C-50 malignant neoplasm of breast	12	3.7%	10	3%	2		0.7%	
C-81 Hodgkin lymphoma	8	2.7%	3	1%	5			1.7%
C-82 Follicular lymphoma	5	2%	5	2%	0			0.0%
Others	101	33.3%	91	30%	10			3.3%
**Origin of the report**	301	100%	264	88%	37			12%
Public	271	90%	237	78%	34			11%
Private	30	10%	27	9%	3			1%

^a^ICD-10: International Classification of Diseases, version 10 [58]

The medical record presented as technical evidence of the need for oncologic medicine was mainly from *SUS* (public origin), both in the *TJ* and in the *TRF (GO)*, representing 90%, while the report of private origin had a frequency of 10% (Table 2).

There were more judicial requests for *NATJUS* opinions (24%) than intermediation by the *CEJUSC* (7%) (Table 3). In *TRF (GO)*, there were no opinions from *CEJUSC*, which was expected because intermediation by the *CEJUSC* is within the scope of the state courts of justice. Included in this analysis were requests for opinions, but they were not necessarily present in all the cases.

**Table 3:** Study legal variables

Variables	All groups		TJGO		TRFGO	
	n	%	n	%	n	%
**Organization**	301	100%	264	88%	37	12%
*CEJUSC*	21	7%	21	7%	0	0%
*NATJUS*	72	24%	52	17%	20	7%
Not specified	208	69%	191	63%	17	6%
**Sponsorship**	301	100%	264	88%	37	12%
Public ministry	138	46%	137	46%	1	0.3%
Public defense	103	34%	68	23%	35	11.6%
Private lawyer	60	20%	59	20%	1	0.3%
**Justification improved QOL**	301	100%	264	88%	37	12%
Yes	141	47%	124	41%	17	6%
No	160	53%	140	47%	20	7%

*QoL* = Quality of life

The justification that the supply of medication would promote improvement in quality of life was used in 47% of the cases in *TJGO* (41%) and *TRF (GO)* (6%) (Table 3).

The preponderant representative, appearing in 47.74% of the cases, was the Public Prosecutor, followed by the Public Defender’s Office with 35.80% of the claims, and finally, Private Lawyers brought 16.45% of the cases. Medical reports originated in the SUS in 97.88% of cases and 2.12% in the private system (Table 3). In addition, there was a significant difference between public defenders and private lawyers (*p* = 0.0007) and public prosecutors and public defenders (*p* = 0.000001).

Table 4 shows Pearson’s correlation between the study variables and length of time in the *TJ* and *TRF(GO).* There was a statistically significant difference (*p* > 0.00001) in the average time between the filing of the action and the granting of judicial protection, whether by preliminary order or decision. In-state courts (88%), the average time was 8.45 + -0.87 days, and in federal courts (12%), 43.5 + -12.90. The actions involved were individual and, for the most part, requesting preliminary relief (72.94%). The delay in federal courts, in large part, is due to requests for expert testimony prior to making a decision. Improving the quality of life of individuals with cancer justified providing the medication in 38.19% of the cases.

**Table 4:** Correlation between study variables and service times at TJGO and TRF-Goiás

Variables	Service time (days)			Service time *TJGO* (days)			Service time *TRF*-Goiás (days)		
	Mean	Standard Deviation	*p*-value	Mean	Standard Deviation	*p*-value	Mean	Standard Deviation	*p*-value
**Gender**									
Male	13.91	16.87	0.74	8.59	5.86	0.85	46.75	38.77	0,32
Female	12.92	20.02		8.78	6.12		32.35	32.12	
**Age**									
0–17	11.62	17.49	0.69	6.83	6.91	0.54	26	36.77	0,73
18–24	4.6	4.72		5.25	5.18		2	NA	
25–34	16.18	24.08		9.67	6.22		35.75	45.58	
35–44	11.36	16.82		7.56	4.6		55	46.67	
45–54	13.76	14.58		10.35	6.85		31.17	26.64	
55–64	10.94	8.7		9.29	6.07		28	14.8	
65 years or older	16.79	25.7		8.17	5.81		54.5	42.46	
**Income**									
Less than 1SM	12.87	14.11	0.96	8.89	4.96	0.904	28.2	25.74	0,48
Greater than 3SM	12.89	15.09		8.14	6.58		29.5	24.19	
Not Specified	13.72	20.13		8.77	6.05		46.4	41.19	
**Sponsorship**									
Public prosecutor	7.86	5.86	0*	7.9	5.87	0.06	4	NA	0,32
Public defense	26.72	29.81		11.18	6.54		40.4	35.36	
Lawyer	9.25	5.33		9.25	5.53		NA	NA	
**Medication**									
temozolamide	12.7	9.93	0.245	11	5.46	0.042*	52	NA	0,121
bevacizumab	14.09	20.33		10.68	7.01		25	41.06	
Rituximab	16.87	31.02		6.84	3.57		82	59.39	
Pazopanib	22.56	25.51		9.5	6.22		44.33	31.2	
pembrolizumab	8.7	5.47		8.7	5.47		NA	NA	
ibrutinib	22.5	35.48		10.14	6.64		109	NA	
pembrolizumab	8.7	5.47		8.7	5.47		NA	NA	
Bortezomide	12.06	11.51		9.88	7.08		49	NA	
Others	9.84	12.48		6.34	5.26		24.9	21.44	
**ICD-10**									
C-90	14.3	12.4	0.0388	10.88	6.83	0.015	45	5.66	0,276
C-71	12.73	10.15		10.95	5.58		52	NA	
C-53	13.7	29.38		5	2.78		3454.58		
C-64	18.79	19.86		9.44	6.6		35.6	25.39	
C-43	13.7	29.38		5	2.78		34	54.58	
C-91	34.2	44.84		9.85	6.54		91	44.8	
C-18	10	9.96		10	9.96		NA	NA	
C-50	20.83	12.56		15.25	8.46		32	14.14	
C-81	11.85	15.24		5	1.73		17	19.49	
C-82	8.25	3.77		8.25	3.77		NA	NA	
Others	9.27	14.13		6.59	4.74		31.17	35.98	
**Origin of the report**									
Public	20.43	12.89	0.701	15.83	5.96	0.726	38.44	35.99	0,69
Private	10.43	12.86		7.6	6.25		53	NA	
**QOL justification**									
Yes	23.508	21.4	0.534	24.49	28.13	0.4024	7	9.18	-
No	20.69	27		20.69	21.4		NA	NA	

^*^p-value < 0.05

There was no significant correlation between gender, age, family income, and origin of the award with the time in *TJGO* and *TRF(GO)*. As well as, there was no association between the justification of provision for improvement of quality of life and the average time for delivery of the jurisdictional object at the significance level (*p* < 0.05) (Table 4).

There was a significant difference between the procedural representatives: between the public defender and lawyer (*p* = 0.0007) and between the public prosecutor and public defender (*p* = 0.000001). In addition, a statistically significant difference was found in medication type and time of care in TJGO (*p* = 0.042) and regarding the ICD-10 described in the process and time of care in TJGO (*p* = 0.015) (Table 4).

## Discussion

In the lawsuits studied, it was identified that most of the claimants for oncological medications were women, representing 54%. Similar studies have found this association between women and health services [62–65]. In addition, this statistic highlights cancer as a family disease and the role of women as formal and informal caregivers of individuals with cancer who could have filed these lawsuits on their behalf [64, 66–69].

The most frequent age groups were people over 55 (52%), with those over 65 representing 30% of the total. In addition, adult and elderly women appear as claimants in other studies [65, 69, 70].

The average income of the plaintiffs in the lawsuits was 1–3 × the minimum wage. Considering that the cost of treating an individual with cancer can exceed R$300.00 per month, the impact of treatment on the family’s finances is high, resulting in suffering and stress [25, 71].

All actions were individual, with the same result found in other studies [44, 65, 72]. The preponderant procedural representative was the Public Prosecutor with 47.74% of the cases, followed by the Public Defender’s Office with 35.80%, and finally, private lawyers with 16.45% of cases. It is important to emphasize that in the requests made by the Public Prosecutor and the Public Defender, the rate of granting preliminary relief is high. Furthermore, preliminary orders have a definitive and irreversible character [73]. Therefore, it could be thought that judicialization is not a tool to promote equity in access to cancer drugs [74].

Regarding the judicial entity, the state of Goiás (88%) was preferred over the federal government (12%) in the present study. In addition, time may have been a determining factor in the choice since, as it turned out, the average time from filing to the delivery of judicial resolution in the state court (8.45 ± 0.87 days) was shorter than in the federal court. (43.5 ± 12.90 days), which could explain the choice. Health-related claims are based on pressing health needs; injunctive relief must be granted to improve life, demonstrating a conceptual mismatch between procedural urgency and medical urgency/emergency [75].

Colombia is also judicializing access to health care through its supreme court, and there has been a growing use of injunctions to protect social rights such as health care indirectly. For example, the judiciary can protect a social right if its non-enforcement causes a violation of a fundamental right, such as life, physical integrity, or human dignity. This understanding is called the doctrine of connection [76] and underpins judicial decisions on the subject in Brazil.

The joint liability of federative entities expands access to judicial intervention. However, the federal government will always be in better financial shape to protect the fundamental right to health concerning the supply of high-cost medicines than states and municipalities [77].

Protecting social rights involves different interpretations according to ideological convictions and the interpreter’s point of view. The judicialization of health, in this context, must be analyzed as a consequence of a hermetic phenomenon involving the need for inter-institutional dialogue and integrated action, whether concerning the current international intellectual property system or policy discussions on research, development, and innovation, whether in price regulation in the pharmaceutical market or assessing which health technologies to include on official lists [78].

The judicialization of health challenges judges to mediate a non-legal conflict between politics and services, impacting public health policies. At the Public Defender’s Office, the challenge is smaller, as this office has practically specialized in demands for comprehensive health care since its implementation in the state in 2016. Before that, through the Chamber of Deputies Technical Assessment in Health *(CATS)* 2009, the state Public Prosecutor analyzed medication demands with a team composed of health professionals [48, 79–81]. These organizations’ specialized case sponsors and free service could explain the greater appearance of public organization sponsors in the study (public defender and public prosecutor) to the detriment of sponsorship by private lawyers and the high number of preliminary orders confirmed in decisions [63, 69].

A medical report must be presented to request medicines in court [43, 48]. In most processes (97.88%), the medical report originated in the public system. In some studies, most reports originated in the private system (38%) [69]. In addition, the medical report or prescription of public origin, issued by a professional from *SUS*, has greater weight as the primary evidence that the health system recognizes the need [73, 82]. Medical reports of public origin combined with the income of the participants demonstrate that judicialization is yet another way for individuals with low family income to access needed medications [6].

One notable aspect of the judicialization of access to medicines is the ability of the magistrate to request an opinion from the Center of Technical Support of the Judiciary (*NATJUS*) before deciding. *NATJUS* aims to provide technical support for decision-making based on scientific evidence in health-related actions. In addition, the Judicial Centers for Conflict Resolution and Citizenship (*CEJUSC*) act to self-composite available rights to resolve conflicts consensually within the state courts of justice [54, 55]. Although not binding, in its use, it can serve as technical support to support judicial decisions [48].

In Goiás, *NATJUS* started its activities in 2012, evaluating public and private health demands, verifying clinical protocols and therapeutic guidelines within the scope of *SUS*, and guidelines for use established by the National Agency for Supplementary Health *(ANS)* [83]. However, despite the recommendations, only 24% of the processes analyzed in the study used the opinion of *NATJUS* and *CEJUSC*.

In cooperation with the Ministry of Health, the National Council of Justice has created a National Opinion Bank, the digital platform (E-NATJUS System) for NATJUS opinions throughout the country. The system is available for consultation by magistrates. The objectives of the platform include reducing the possibility of conflicting judicial decisions on issues related to medicines and treatments and the concentration in a single database to avoid the formalization of requests whose treatments are not recommended by official health agencies [41, 84]. In Goiás, in 2020, there were 2016 consultations and 2081 opinions formulated, originating from requests from 1st-degree magistrates, mostly [83].

The most prevalent neoplasm in the lawsuits was brain cancer, followed by multiple myeloma. In the study by CERVI et al. [44], in 2020, in the state of Rio Grande do Sul and by Santos (2021) [65] in São Paulo, the most frequent neoplasm was also multiple myeloma.

Of the most requested drugs in the study, bevacizumab is indicated for metastatic colorectal cancer, advanced or metastatic lung, metastatic breast, renal, ovarian, and metastatic cervical cancer. In addition, bevacizumab was considered the agent of choice in clinical protocols and therapeutic guidelines for managing diabetic retinopathy, glaucoma, and neovascular age-related macular degeneration (AMD) in 2018. However, after the non-renewal of off-label use by the resolution of the collegiate board of *ANVISA*, *CONITEC* in 2021 recommended other drugs for this purpose (aflibercept and ranibizumab) [55, 85].

The addition of bevacizumab as a molecular therapy to palliative chemotherapy in patients with recurrent, persistent, or metastatic cancer was associated with a 3.7-month improvement in overall survival. Even so, this improvement would not be considered cost-effective in the United States, given the price of the current treatment [86]. Indeed, in the study by GOMES et al. (2021) [25], the total average cost of treatment was R$531.87, estimated per procedure session.

In the present study, in most of the legal requests for bevacizumab, it was prescribed for AMD when it was legal for off-label use [69, 87].

The drug temozolomide was, together with bevacizumab, the most requested. However, *CONITEC*, in a 2014 recommendation report on temozolomide for the adjuvant treatment of patients with High-Grade Gliomas, stated that using the temozolomide as an adjuvant in the treatment of brain neoplasia increased survival but decreased quality of life. Thus, the basis for improving the quality of life for access to high-cost oncological drugs is a paradox that deserves reflection since the administration of drugs in individuals with cancer can often worsen the desired quality of life in patients with cancer due adverse drug reactions [88].

Concerning the reasons for the judicial processes, 47% identified quality of life as the justification for requesting cancer medication. There are several concepts of quality of life. The World Health Organization (WHO) suggests this complex concept as a subjective way for individuals to perceive their lives, defining their goals and perspectives, including their satisfaction with life, cultural context, social, environmental, and value systems in which they are inserted [89, 90]. Notably, in 1995, the Food and Drug Administration (FDA) included the need to assess the quality of life for the approval of oncological drugs [91].

Considering that medical and pharmaceutical practice shows that oncological drugs cause many adverse reactions, are difficult to adapt to, and in most cases reduce the quality of life of patients, there seems to be a terminological confusion between improvement in quality of life and increased survival [92].

In order to be considered beneficial, an increase in survival must be accompanied by improved quality of life. Otherwise, it would prolong suffering and pain for the individual and their family. Indeed, prolonging life must respect the interests and dignity of the individual in question as an active participant in their care process, overcoming professional interests of trying to extend life at any cost with experimental treatments, prolonged hospital stays, and invasive procedures that increase the suffering they already experienced, in addition to being resource-intensive [93–95].

The judiciary cannot avoid the debate about resource allocation in health and its relationship with the principles of universality, equity, and integrality that guide *SUS*. However, it must also weigh this social protagonism with individual decisions that significantly impact the entire State structure [77]. For granting access to medications and other health technologies through judicial protection, the cost-utility analysis (CUA) should be suggested as an alternative. The CUA is more suitable for health-related quality of life, focusing mainly on the quality of the health outcome produced or avoided and introducing the QALY concept – quality-adjusted life years [96]. QALY is a cost-to-effectiveness threshold calculation that aims to maximize years and quality of life from a utilitarian perspective.

*CONITEC* suggests that the economic evaluation and cost-effectiveness parameters for approving new technologies for *SUS* should be based on quality-adjusted life years (QALY). However, a cost-effectiveness threshold and the QALY cannot be obstacles that hinder other relevant decision-making criteria when it comes to adding new technologies to *SUS* approval lists [97].

Indeed, the experience of countries like Australia (Medical Service Advisory Committee—MSAC), Canada (Canadian Agency for Drugs and Technology in Health—CADTH), and the United Kingdom (National Institute for Health and Clinical Excellence—NICE) in health technology assessment may represent an important starting point for CONITEC in the adoption of cost-effectiveness thresholds. However, it should be noted that these value definitions are context-specific, depending on local wealth, health system characteristics, availability, ability to pay, and social preferences. Therefore, such issues must be considered while issuing the regulations in art. 19-Q, §3, of Law No. 8.080/90 [98].

Indeed, health benefits can be maximized, including cancer drugs, through cost-effectiveness (or utility) weightings based on quality of life, achieving the ideals of justice without abdicating the primary public interest [73].

## Conclusions

The lawsuits analyzed in the study were brought by women aged over 55 years, of low income, and sponsored by public prosecutors and public defenders to request, mainly, medications prescribed for multiple myeloma and brain cancer that are not included on SUS official lists, with few requests for opinions from *NATJUS* and mediation from *CEJUSC*.

Using quality of life to justify these medication requests presents a dubious understanding when it comes to increased survival. The apparent confusion disregards the deleterious effects of oncological therapy and its negative impact on quality of life. Increased survival is important and should not be neglected in this assessment if it improves an individual’s quality of life, but the erroneous use of non-synonymous expressions, quality of life, and increased survival should be avoided.

There are many financial challenges to fully implementing oncologic care. *SUS* has been underfunded since it was created 34 years ago, with federal budget cuts below the minimum required to meet constitutional guarantees. The market is overvalued, and state power has been reduced. Only broad, profound changes in the current economic model and social structures that break neoliberal paradigms will ensure medium and long-term sustainability.

It should be noted that greater profits are guaranteed to the pharmaceutical industry through expanded access to medications by lower-income patients. Even if severe questions of inequity are not raised, it can be argued that industry profits are driving this process, not access to medications.

Finally, the judicialization of health indicates that specific health needs arise even with defined and equitably implemented public policies. These needs must be considered for the state’s effective provision of public services to guarantee the right to health.

The present work has strengths and limitations. One of the strengths is being the first study in the central region of Brazil and having a relatively large sample size. The size may suggest the generalizability of the results obtained. However, as a possible limitation, cancer stages and previous treatment protocols were not considered relevant, as they do not interfere with the court’s response.

**Table:** 

**Frequency table:**			
Decision			
Quality of life	< Mean		> Mean
No	32		14
Yes	83		41
Pearson’s Chi-squared test			
Data: Table			
X-squared = 0.10602, df = 1, *p*-value = 0.7447			
There was no association between time and quality of life			
**Welch Two Sample t-test:**			
Data: Time by Quality of life			
t = -0.69995, df = 103.67, *p*-value = 0.4855			
Alternative hypothesis: true difference in means between the No group and Yes group is not 0			
95 percent confidence interval:			
-10.780632		5.155807	
Sample estimates:			
Mean in No group		Mean in Yes group	
20.69565		23.50806	

## Data Availability

All data generated or analyzed during this study are available from the corresponding author on reasonable required.

## Abbreviations

SUS: Brazilian Public Health / *Sistema Único de Saúde*
CONEP: National Ethics Committee
FDA: Food and Drug Administration
WHO: World Health Organization
CF: Federal Constitution / *Constituição Federal*
INCA: National Cancer Institute / *Instituto Nacional do Câncer*
UNACON: High-Complexity Oncology Care Unit / *Unidade de Assistência de Alta Complexidade em Oncologia*
CACON: High Complexity Oncology Care Center / *Centro de Assistência de Alta Complexidade em Oncologia*
MS: Ministry of Health / *Ministério da Saúde*
DDT: Diagnostic and Theraputic Guiidelines / *Diretrizes* *Diagnósticas* *e* *Terapêuticas*
PCDT: Clinical Protocols and Therapeutic Guidelines / *Protocolos* *Clínicos* *e* *Diretrizes* *Terapêuticas*
PIB: Gross Domestic Product / *Produto* *Interno* *Bruto*
ANVISA: Brazilian National Health Surveillance Agency / *Agência* *Nacional* *de* *Vigilância* *Sanitária*
TJGO: Goias State Court of Justice / *Tribunal* *de* *Justiça* *do* *Estado* *de* *Goiás*
TRF: Regional Federal Court / *Tribunal* *Regional* *Federal*
CEMAC: Center for High-cost Medications / *Central* *de* *medicamentos* *de* *alto* *custo*
PROJUDI: Digital Judicial Process / *Processo* *Judicial* *Digital*
PJE: Electronic Judicial Process / *Processo* *Judicial* *Eletrônico*
CEAF: Specialized Pharmaceutical Assistance Component / *Componente* *Especializado* *da* *Assistência* *Farmacêutica*
NATJUS: Center for Judicial Technical Support / *Núcleo* *de* *Apoio* *Técnico* *do* *Judiciário*
CEJUSC: Judicial Centers for Conflict Resolution and Citizenship / *Centros* *Judiciários* *de* *Solução de* *Conflitos* *e* *Cidadania*
CID: International Classification of Diseases / *Classificação* *Internacional* *de* *Doenças*
CATS: Chamber of Technal Evalutation in Health / *Câmara* *de* *Avaliação* *Técnica* *em* *Saúde*
DP: Public Defender / *Defensoria* *Pública*
MP: Public Prosecutor / *Ministério* *Público*
DMRI: Age related macular degeneration / *Degeneração* *Macular* *Relacionada* *à* *Idade*
ACU: Cost-effectiveness analysis / *Análise* *de* *custo-efetividade*
QV: Quality of life / *qualidade* *de* *vida*
QALY: Quality-Adjusted Life Years
SM: National minimum wage (Salário-Mínimo)
